# Panoramic Radiography vs. CBCT in the Evaluation of the Maxillary Third Molar Roots

**DOI:** 10.3390/medicina59111975

**Published:** 2023-11-09

**Authors:** Marcia Almeida-Marques, Mara Magnoler Sampaio Ingold, Alberto Ferreira da Silva-Junior, Ademir Franco, José Luiz Cintra Junqueira, Anne Caroline Oenning

**Affiliations:** 1 Division of Oral Radiology, Faculdade São Leopoldo Mandic, Centro de Pesquisas São Leopoldo Mandic, Rua José Rocha Junqueira 13, Ponte Preta, Campinas 13045-755, Brazil; 2 Division of Forensic Dentistry, Faculdade São Leopoldo Mandic, Centro de Pesquisas São Leopoldo Mandic, Rua José Rocha Junqueira 13, Ponte Preta, Campinas 13045-755, Brazil

**Keywords:** third molar, maxilla, cone-beam computed tomography, panoramic radiography

## Abstract

*Background and Objectives:* A comprehensive understanding of the position of third molar roots and adjacent structures, such as the maxillary sinus (MS), is essential for safe extractions. Diagnostic imaging plays a fundamental role in achieving accurate treatment planning. This study aimed to compare panoramic radiography (PR) and cone-beam computed tomography (CBCT) for the evaluation of maxillary third molar roots and their relationship with the MS. *Materials and Methods:* Two trained radiologists evaluated third molar images. The number of roots, morphology (fused/conical, divergent, dilacerated, or atypical), and their relationship with the MS in PR and CBCT were registered. Descriptive and inferential statistics were performed using the weighted Kappa test. *Results:* Regarding the number and morphology of the roots, Kappa values showed moderate (κ = 0.42) and fair agreement (κ = 0.38), respectively. Regarding the proximity with the MS, most of the roots showed close contact (30.6%), or 1/3 of root superimposition (35%), in PR evaluation, while in CBCT, the third molars were in contact with the MS floor (32%), and with alveolar domes (27.2%). *Conclusions:* PR is a moderately reliable image technique to identify the number of roots and root morphology of maxillary third molars. PR, however, does not provide any radiographic signs that clearly indicate the anatomical relationship between the maxillary third molar roots and the maxillary sinus detected in CBCT images.

## 1. Introduction

The extraction of third molars is a common procedure in the routine of oral and maxillofacial surgery [1,2]. More specifically, maxillary third molar extraction has low morbidity and a low level of complexity because the maxillary bone is less dense and more flexible than the mandible and has no vital neurovascular structures adjacent to the extraction site [3]. Despite the low risk, the surgery requires substantial planning and surgical skills, as intercurrences may occur [3]. A prospective multicenter study showed that oroantral communication is a common intercurrence in maxillary third molar extractions, occurring in 13% of the cases [4]. Other examples include alveolitis, infection, hemorrhage, damage to adjacent teeth, tooth displacement, and bone fracture [5].

Assessing image exams prior to third molar extractions is essential. Radiographs can help surgeons visualize the maxillary sinus (MS), its anatomical limits, and the relationship with the roots of posterior teeth, for instance [6]. Panoramic radiography (PR) is frequently performed for the preoperative planning of third molar extractions. This type of image allows an overview of tooth position, including the angle of third molars, their relationship with second molars, and impaction [7]. The superimposition of maxillofacial structures on a two-dimensional plane, however, may hamper image visualization and could jeopardize treatment planning. In contrast, three-dimensional examination via cone-beam computed tomography (CBCT) provides a multiplanar visualization that is free of distortion, magnification, and overlapping [8]. Studies have investigated the interface of CBCT and maxillofacial surgery in different ways, namely to assess third molar impaction [9], condylar displacement after orthognathic surgery [10], and to predict neurovascular damage [11]. When it comes to maxillary third molars, studies have a major interest in the effects of marginal bone loss induced by impacted teeth [12], the relationship (proximity) between maxillary third molars and MS [13], and the associated risks of oroantral communication and facial swelling [14], for instance.

Comparisons between PR and CBCT have been developed in the scientific literature to understand the effects of different image exams on the clinical decision-making process as well as on the outcomes of maxillofacial surgeries [15]. An existing scientific gap, however, persists when maxillary third molars are considered. In other words, most of the comparative studies between image techniques have addressed the mandibular third molars, given their complexity in routine oral surgeries. The current scientific literature shows that a proper assessment of the roots of maxillary third molars, considering their number, morphology, and relationship with the MS, is imperative to avoid trans- and postoperative intercurrences [16]. Thus, this study aimed to compare the diagnostic value of PR and CBCT for the assessment of the roots of maxillary third molars and their relationship with the MS.

## 2. Materials and Methods

### 2.1. Study Design/Sample

This was a cross-sectional observational study approved by an Institutional Committee of Ethics in Human Research (protocol: 3.974.124). The sample consisted of PR and CBCT scans of 206 maxillary third molars from 137 patients, males and females, older than 15 years, who had taken both PR and CBCT exams at the dental radiology clinic of a private dental school. Since this was a retrospective study, no patient was exposed to ionizing radiation for research purposes. They manifested consent for image acquisition in their first appointment in the radiology clinics and started treatment at the university. With an image database built, research was possible by revisiting the patients’ files. The sample size was considered based on data availability and selected for convenience. The study of Neves et al. (2012) [14] served as a reference for a minimum sample size (n = 72 individuals, 142 third molars). Image acquisition was retrospective from an existing radiographic database and was justified because the patients were scheduled for maxillary third molar extractions. The time between PR and CBCT was less than 30 days apart. The acquisition of a CBCT scan after an initial PR relied on the complexity of the case (complex cases of third molar extraction required a 3D visualization using CBCT instead of PR). Patients with incomplete root formation and pathological anomalies, such as cystic/tumoral lesions associated with the maxillary third molars, were excluded from the study.

### 2.2. Image Acquisition and Evaluation

Digital panoramic radiographs obtained with an Ortopantomograph OP200D device (Instrumentarium DentalTM, Tuusula, Finland) operating at 66 kVp, 8 mA, and 14 s of exposure, were collected. Tomographic images were obtained from two devices: i-CAT (Imaging Sciences International, Hatfield, PL, USA), using a protocol of 120 kVp, 36 mAs, FOV of 13 × 16 cm and voxel of 0.25 mm, and OP300 (Instrumentarium DentalTM, Tuusula, Finland) with 85 kVp, 13 mAs, FOV of 8 × 15 cm and 0.25 mm voxel, besides the use of FOV 13 × 15 cm with 0.32 mm voxel. Panoramic radiographs were exported in JPEG format and analyzed with Image J Software v1.53j (National Institute of HealthTM, Bethesda, MD, USA). Tomographic images as DICOM files were dynamically analyzed in axial, sagittal, and coronal views (XoranCat—Xoran Technologies, Ann Arbor, MI, USA, and OnDemand 3D—CyberMed Inc., Seoul, Republic of Korea) on a 23 inch LCD monitor with 1920 × 1080 resolution under controlled light conditions. Whenever necessary, zooming, adjustments of brightness or contrast, and reformatting of selected planes were used.

Two radiologists were trained to perform image assessment after receiving an explanation of the required image analysis method. Subsequently, interpretation and reproduction were tested through a pilot using 20 pairs of images that did not compose the main sample of the study.

PR images were assessed first. To avoid bias from image memorization, tomographic images were analyzed only thirty days later. Both evaluations were performed separately by the two trained radiologists, randomly and blindly, regarding the patient’s clinical data. Next, the outcomes of the evaluations of both radiologists were compared. Disagreements were solved first by discussion and, if necessary, by consulting a third author for a decision.

**Figure 1:**
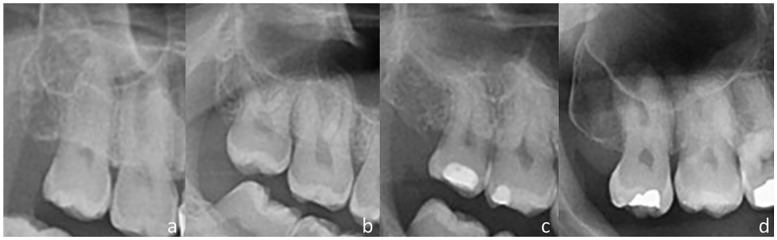
Root morphology classification: (**a**) Fused/conical anatomy; (**b**) divergent; (**c**) dilacerated; and (**d**) atypical shape.

The patient’s gender, age, and number of upper third molars (if unilateral or bilateral) were recorded. The number of roots and root morphology (fused/conical, divergent, curve, and atypical) were documented (Figure 1). The diagnosis of root fusion relied on the involvement of at least two-thirds of the root. Root dilaceration was considered when at least one root presented a marked curvature of the root. Dilaceration of multiple roots from the same tooth was considered a single finding.

**Figure 2:**
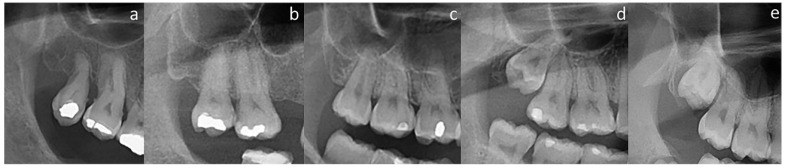
Relationship between roots and MS on PR: (**a**) MS floor above the root(s); (**b**) apices in close contact with MS floor; (**c**) MS floor overlapping on 1/3 of the roots; (**d**) MS floor overlapping on 2/3 of the roots; and (**e**) MS floor overlapping on the furcation.

**Figure 3:**
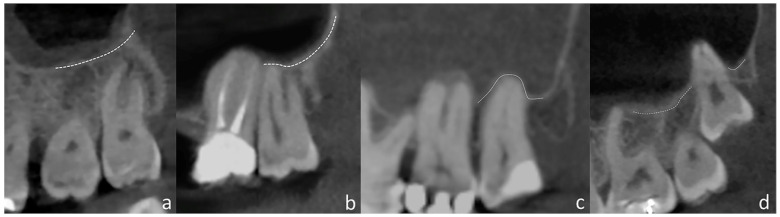
Relationship between roots and MS on CBCT: (**a**) apices distant from the MS floor; (**b**) MS floor touching the roots; (**c**) presence of alveolar domes; and (**d**) absence of bone covering.

Since PR has superimposition and distortion, while CBCT scans do not, they are interpreted differently. In PR, the localization of the maxillary third molar root apices relative to the MS floor was evaluated according to the study by Pourmand et al. [2] (Figure 2). Figure 3 demonstrates the various patterns of relationship between the root apices of maxillary third molars and the MS floor on CBCT images.

### 2.3. Statistical Analysis

A descriptive analysis was performed. Absolute and relative frequencies were used for categorical variables, while mean, standard deviation, and maximum/minimum values were used for continuous variables. Also, weighted Kappa values were calculated to determine the level of agreement between PR and CBCT findings, considering a 95% confidence interval. The interpretation of κ values was: κ < 0.00 indicated poor agreement, 0.0–0.19 indicated slight agreement, 0.2–0.39 indicated fair agreement, 0.4–0.59 indicated moderate agreement, 0.6–0.79 indicated substantial or good agreement, and >0.8 indicated excellent or almost perfect agreement. All the analyses were conducted with R statistics (R Foundation, Vienna, Austria).

## 3. Results

Regarding the analysis of root number, there was an overall moderate agreement between PR and CBCT in 54.3% of teeth (κ = 0.4241) (Table 1). Considering CBCT as the reference image exam, an agreement was found in thirty-nine (18.9%) of the fifty-six single-rooted teeth, fourteen (6.8%) of the thirty-one teeth with two roots, and fifty-nine (28.6%) of the one hundred and eleven teeth with three roots, while no agreement was found in the eight teeth with four roots. Image assessment in PR led to two teeth classified as single-rooted, two teeth classified as two-rooted, and four teeth as three-rooted.

Overall agreement for root morphology was 73.7% (κ = 0.38, fair agreement) (Table 2). Of the 143 teeth classified as fused/conical roots via CBCT, agreement was found in 130 teeth (63.1%). Of the 31 teeth with divergent roots, agreement was detected in 11 teeth (5.3%). Of the 26 teeth with dilaceration, agreement was detected in 10 (4.8%). Finally, one (0.5%) out of the six roots with atypical anatomy showed agreement between CBCT and PR.

Because of the different visualization between 2D (PR) and 3D (CBCT) images, an agreement analysis was not applied to evaluate the anatomical relationship between the MS and the roots of the maxillary third molar (Table 3). Descriptive data on this topic showed that out of the 45 teeth with apices distant from the MS floor (classified using CBCT), 34 (75.6%) were classified on PR as having the MS floor above the roots. Of the 66 teeth classified as having the MS floor touching the third molar roots, 84.8% were classified on PR either as having apices touching the sinus floor (n = 28, 42.4%) or exhibiting 1/3 of the roots overlapping the MS floor (n = 28, 42.4%). The presence of alveolar domes on CBCT images (n = 56) was also classified as having apices in close relationship with the MS floor (n = 17, 30.4%) and 1/3 of the roots overlapping (n = 23, 41.1%) the MS floor in PR. The absence of bone covering the MS roots was detected on CBCT images of 39 teeth (19%). In PR, these findings were classified as twenty teeth (51.3%) having 1/3 of root overlapping, eight teeth (20.5%) having apices in close contact with the MS floor, eight teeth (20.5%) with 2/3 of root overlapping the MS floor, and three teeth (7.7%) overlapping down to the furcation region.

## 4. Discussion

The present study provides evidence that a 2D, low-dose radiographic technique (PR) is relatively reliable in evaluating the number and morphology of roots in maxillary third molars. However, in cases where knowledge of root position relative to the MS is necessary before the extraction of maxillary third molars, CBCT should be recommended. This situation is especially relevant in patients that have alveolar extensions of the maxillary sinuses, posterior teeth with long roots, or simply patients that have a close relationship between teeth and MS. It is the inherent knowledge and perception of surgeons that will dictate the need for more detailed anatomical visualization of the patient. In addition to their knowledge, this study might support the decision-making process in daily clinical practice with evidence regarding the differences between PR and CBCT on the visualization of third molars and adjacent dentomaxillofacial structures.

The correct identification of the number of roots is an important aspect to consider before tooth extraction, as it can influence the level of complexity of maxillary third molar extraction since the risk of root fracture during extraction increases with teeth that have more roots [17]. In this study, a moderate level of agreement between CBCT and PR was observed when assessing the number of roots, with a higher number of roots identified using CBCT. The highest agreement was mainly found in the “single-rooted” category (69.6%), which suggests limitations of PR to detect multiple roots in maxillary third molars. In this regard, the present findings are consistent with previous research demonstrating the limited accuracy of PR to assess the number of roots [18]. Likewise, similarity was found with other studies that compared PR to clinical findings, in which a lower number of maxillary third molar roots was detected in PR [17]. The difficulty in evaluating roots in this region using PR comes from the anteroposterior superimposition of roots [19]. According to Lubbers et al. [20], if teeth have three or four roots, evaluation with PR becomes difficult due to the overlapping of at least two of them. It must be noted, however, that the maxillary third molars with four roots were the least prevalent (3.9%) in our study, while the three-rooted third molars were the most common (53.9%). This pattern of roots reflects the most common anatomy of third molars, which resembles the first or second molars. In general, these teeth have two buccal roots and one palatal. On the one hand, the palatal root is more visible because of its longer and more robust shape, while the buccal roots are smaller and more difficult to visualize in PR.

The limitations of PR in assessing root morphology have already been reported in the literature [17,18]. Although our results revealed differences between the evaluated categories of root morphology, we observed an overall agreement of 70% between CBCT and PR. However, according to Kappa statistics, the level of agreement was merely fair. The statistical analysis revealed that the highest percentage of agreement (90.9%) was observed in cases of fused/conical roots, indicating that PR has a greater potential for identifying this type of root morphology in maxillary third molars. When considering other categories, an agreement below 40% was detected. The fused roots (multiple) or tapered conical roots (single) were combined (unified) in this study because of the difficulty in distinguishing them in image exams. The unified group was prevalent on CBCT (n = 143, 69.4%), followed by the group with divergent roots (n = 31, 15%), which is consistent with previous studies [21]. Different results were observed in a study with the Thai population, in which 50.9% of the extracted maxillary third molars had separated roots and 45.7% had fused or tapered roots [22]. Studies, however, have used different methods of root morphology visualization concerning the current study, namely PR, periapical radiographs, and clinical and laboratory findings. A limitation relies on the scarce literature to enable better comparisons with the present findings when it comes to maxillary third molars and their visualization through CBCT.

Root dilaceration was the third most common morphologic characteristic in this study and was identified in 14 (6.8%) and 26 teeth (12.6%) via PR and CBCT, respectively. This finding supports previous studies highlighting the fact that mesial or distal root bends are observed on PR [23]. Nevertheless, it is difficult to identify dilaceration on PR when it occurs in the buccal and palatal directions, which consequently increases the possibility of diagnostic errors [23]. CBCT is indicated by some authors as the most accurate exam to assess the angulation and direction of inclination of roots with dilaceration. In practice, this tool can provide valuable information to support clinical decisions related to surgical planning [24]. In the present study, we have not assessed specifics of root dilaceration, such as root angles, for instance. Future studies in the field could be designed to include metric analyses using CBCT.

Few maxillary third molar roots were recorded as having “atypical morphology”, and all cases identified by CBCT (n = 6) were related to hypercementosis. This condition is, indeed, not a common finding, with a prevalence ranging from 0.6% to 10.8% [25]. Hypercementosis is most frequently found in mandibular premolars [26] and molars [27]. Studies have demonstrated the insufficient performance of PR to detect hypercementosis [28]—especially because in 2D images only mesiodistal extensions can be visualized. Few studies have evaluated hypercementosis using CBCT [27,29], and even fewer have assessed maxillary third molars [29]. Lack of a proper diagnosis of hypercementosis before a surgical intervention increases complexity due to the greater anchorage of the tooth in the bone and the possibility of tooth or bone fracture.

A correct treatment plan requires preoperative knowledge of the relationship between the maxillary third molar and the MS. Thus, a careful evaluation of image exams before surgery should be performed, since the roots may be in contact or even projected into the sinus [30,31]. In the present study, among the teeth classified based on CBCT as having apices distant from the MS floor, most (75%) were classified on PR as having the sinus floor above the roots, which points out a similarity in the diagnosis. On the other hand, teeth classified in CBCT with roots in contact with the sinus floor (touching or alveolar dome) were classified in PR mostly as having apices close to or superimposed with the sinus floor. When it comes to surgical complexity, the situation that represents the highest risk is the absence of bone contour/coverage around the apices. This situation was detected in 39 teeth using CBCT. In PR, most of these teeth presented a superimposition of the MS floor over 1/3 of the third molar roots. This agrees with the current literature that points out a higher diagnostic reliability of PR when the roots are below (“distant”) the floor of the MS and a lower agreement between PR and CBCT when the roots are in contact or projected above the sinus floor [32,33,34]. The authors explain that the superimposition of anatomical structures may lead to misinterpretation of root protrusion into the sinus cavity [32]. From a practical point of view, the safer situation for the surgeon is when a distance exists between the MS floor and the maxillary third molar roots. Because of the inherent proximity between the maxillary third molar and the MS in the posterior region of the maxilla, a safe zone (distance) might not be frequent in patients scheduled for extractions. In this case, the surgeon might face a better situation when there is a superimposition of images of the maxillary third molar roots and the sinus floor—instead of a protrusion of the roots into the MS. Extractions under this condition might have a higher risk of oroantral communication.

The most prevalent relationships between the MS and roots of the third molars observed on CBCT exams were: (1) the MS floor touching the roots; and (2) the presence of alveolar domes (when there is a contour of the MS floor over the third molar apices). Therefore, in most of the cases, the third molars were close to the maxillary sinus, posing a higher risk during surgery. Thus, when the surgeon suspects a potential proximity between the maxillary third molar roots and the MS based on PR findings, CBCT can serve as an additional tool to enhance the predictability of the procedure and increase the safety of the surgery. By providing a comprehensive evaluation of the real relationship between these structures, CBCT can help identify potential risks and enable the surgeon to make optimal decisions regarding the surgical approach [8]. In this context, it is worth mentioning that recent technology has made possible a more realistic view of the anatomical structures of interest to surgeons, especially using 3D image acquisition and image segmentation. Researchers have highlighted benefits that cover the correct identification of the third molar position [35], as well as studying dental lesions such as root resorption [36].

It is a common understanding in the scientific literature that CBCT overcomes the limitations of PR by providing multiplanar views free of distortion and superimposition. However, it is worth noting that this imaging modality presents a higher biological cost (radiation dose), which requires the use of set criteria for its indication. To obtain useful information for diagnosis and optimize the radiation dose by preventing the harmful effects of ionizing radiation associated with CBCT, the European project DIMITRA (Dentomaxillofacial Pediatric Imaging: an investigation towards low-dose radiation-induced risks) proposed the progression of the radioprotection principles ALARA (“as low as reasonably achievable”) and ALADA (“as low as diagnostically acceptable”) to ALADAIP (“as low as diagnostically acceptable being indication-oriented and patient-specific”) [37]. Thus, ALADAIP is based on basic, already consolidated radioprotection principles, such as justification, and recommends that all CBCT examinations should be optimized considering the diagnostic task and the exposed individual, especially when it comes to children and adolescents [38]. In this context, it is worth noticing that most of the patients who are scheduled for preoperative imaging examinations regarding third molar extractions are young. Hence, balancing the cost-benefit of CBCT as an imaging diagnosis tool for third molar extraction is fundamental before planning surgeries exclusively using PR or directly via CBCT. What surgeons without knowledge of oral radiology usually think is that a tridimensional image technique, such as CBCT, would lead to a more accurate diagnosis and treatment plan, regardless of the clinical justification. However, this way of thinking ignores the patient-specific indication of the image exam. In other words, a PR could be enough for several applications in the clinical routine, while CBCT should be an exception for young individuals—becoming the imaging technique of choice only when indispensable.

The current literature addressing the variables that we assessed in this study uses different classifications, tooth groups, and image exams. Moreover, there were only a few studies evaluating the number and morphology of maxillary third molar roots, comparing PR and CBCT. Thus, the contribution of the present study is highlighted. The main limitations of the present study include the lack of metric analyses of root dilaceration and proximity with the MS (1) and the lack of clinical comparisons with the image findings (2). Future studies in the field could overcome the first limitation by adding methodological steps to guarantee a full metric analysis of the angles formed in roots with dilaceration. This procedure could benefit from the inherent metric tools available in image viewers, for instance. With measured angles, classification systems could be established, and PR and CBCT could be compared for their capability to distinguish roots with certain levels of dilaceration. The second limitation could be approached and overcome with studies dedicated to integrating clinical outcomes (trans- and postoperative) with image findings. In this context, the clinical aspect of an extracted third molar could confirm (or not) the image findings observed in PR and CBCT. By doing so, the extracted third molars could serve as a gold standard (reference) for the anatomical aspects to be compared between PR and CBCT.

## 5. Conclusions

PR is a relatively reliable image technique that is useful to visualize and classify the number and morphology of maxillary third molar roots, especially single-rooted teeth and fused/conical-shaped roots. PR, however, might not be accurate enough to enable a proper visualization of the anatomical relationship between the roots of the maxillary third molars and the MS. Clinicians should keep in mind PR as the proper tool to assess basic features of the maxillary third molars, such as the number of roots, but when there is a suspicion of proximity between the roots of the maxillary third molar and the MS floor (posing a higher surgical risk), CBCT could be considered.

## Figures and Tables

**Table 1:** Teeth distribution according to the number of roots, evaluated by panoramic radiography and cone-beam computed tomography (CBCT).

Panoramic Radiograph	Cone-Beam Computed Tomography (CBCT)				Total
	1 Root	2 Roots	3 Roots	4 Roots	
1 root	39 (18.9%)	14 (6.8%)	13 (6.3%)	2 (1.0%)	68 (33.0%)
2 roots	13 (6.3%)	14 (6.8%)	39 (18.9%)	2 (1.0%)	68 (33.0%)
3 roots	4 (1.9%)	3 (1.5%)	59 (28.6%)	4 (1.9%)	70 (34.0%)
4 roots	0 (0.0%)	0 (0.0%)	0 (0.0%)	0 (0.0%)	0 (0.0%)
Total	56 (27.8%)	31 (15.0%)	111 (53.9%)	8 (3.9%)	206 (100.0%)

Percentages are concerning total teeth (n = 206). Agreement = 54.3%; weighted Kappa = 0.4241 (95% CI: 0.3331–0.5150).

**Table 2:** Teeth distribution according to root morphology, assessed by panoramic radiography and cone-beam computed tomography (CBCT).

Panoramic Radiograph	Cone-Beam Computed Tomography (CBCT)				Total
	Fused/Conical	Divergent	Dilacerated	Atypical	
Fused/Conical	130 (63.1%)	20 (9.7%)	11 (5.3%)	5 (2.4%)	166 (80.6%)
Divergent	9 (4.4%)	11 (5.3%)	4 (1.9%)	0 (0.0%)	24 (11.6%)
Dilacerated	4 (1.9%)	0 (0.0%)	10 (4.8%)	0 (0.0%)	14 (6.8%)
Atypical	0 (0.0%)	0 (0.0%)	1 (0.5%)	1 (0.5%)	2 (1.0%)
Total	143 (69.4%)	31 (15%)	26 (12.6%)	6 (2.9%)	206 (100%)

Percentages are concerning total teeth (n = 206). Concordance = 73.7%; weighted Kappa = 0.3811 (CI 95%: 0.2424–0.5195).

**Table 3:** Teeth distribution according to the relation between teeth roots and MS floor, assessed by panoramic radiography and cone-beam computed tomography (CBCT).

Panoramic Radiograph	Cone-Beam Computed Tomography (CBCT)				Total
	Apices Distant from the Floor	MS Floor Touching the Roots	Presence of Alveolar Dome	Absence of Bone Covering	
MS floor above the roots	34 (75.6%)	4 (6.1%)	0 (0.0%)	0 (0.0%)	38 (18.4%)
Apices—MS floor in close contact	10 (22.2%)	28 (42.4%)	17 (30.4%)	8 (20.5%)	63 (30.6%)
MS floor overlapping 1/3 root	1 (2.2%)	28 (42.4%)	23 (41.1%)	20 (51.3%)	72 (35.0%)
MS floor overlapping 2/3 root	0 (0.0%)	4 (6.1%)	8 (14.3%)	8 (20.5%)	20 (9.7%)
MS floor overlapping furcation	0 (0.0%)	2 (3.0%)	8 (14.3%)	3 (7.7%)	13 (6.3%)
Total	45 (100%)	66 (100%)	56 (100%)	39 (100%)	206 (100%)

Percentages are concerning total teeth (n = 206). MS: maxillary sinus.

## Data Availability

Data are contained within the article.
